# Total Hip Arthroplasty (THA) for Femoral Neck Fractures: Comparison between Standard and Dual Mobility Implants

**DOI:** 10.3390/geriatrics6030070

**Published:** 2021-07-07

**Authors:** Riccardo L. Alberio, Mattia Rusconi, Loris Martinetti, Diego Monzeglio, Federico A. Grassi

**Affiliations:** 1 Orthopaedics and Traumatology Unit, Hospital “Maggiore della Carità”, 28100 Novara, NO, Italy; riccardo.alberio@gmail.com (R.L.A.); diego.monzeglio@libero.it (D.M.); federico.grassi@med.uniupo.it (F.A.G.); 2 Department of Health Sciences, University of East Piedmont, 28100 Novara, NO, Italy; lorismartinetti@hotmail.it

**Keywords:** dual mobility cup, total hip arthroplasty, femoral neck fracture, dislocation, elderly patients

## Abstract

The purpose of this retrospective study is to compare the short-term clinical and radiological results between standard and dual mobility THA for femoral neck fractures (FNF) in older patients. The hypothesis is that the dual mobility cup (DMC) has the same outcomes but a lower dislocation rate than the standard THA. The study population included 56 patients (mean age 77.7 years, range 71–85) that underwent THA for displaced FNF. Patients were divided in two comparable groups for baseline characteristics (age, sex and comorbidities): 28 patients underwent THA with a standard cup (SC) and 28 THA with DMCs. The clinical records and radiograms were reviewed to search relevant data in their postoperative history. Two postoperative dislocations occurred in the SC group and none in the DMC group. At an average follow up of 23 months (12–40), 48 patients were available for the final evaluation. The WOMAC score for all patients averaged 6.26 (0–46) and was slightly better in the DMC group (4.94 vs. 7.58; *p*-value = 0.41); scores were significantly better in presence of neurological comorbidities (*p*-value = 0.04), in the absence of diabetes (*p*-value = 0.04) and in the case of psychiatric disorders (*p*-value = 0.02). Radiographic evaluation at one year showed signs of osteointegration in 42/48 (87.5%) acetabular components (20 DMCs, 22 SC). According to our experience, DMCs proved to be a valid option for the treatment of displaced FNF in older patients, since it allowed them to achieve short-term outcomes comparable to conventional THA, while decreasing the incidence of postoperative dislocations.

## 1. Introduction

Dislocation is one of the most frequent complications of total hip arthroplasty (THA), with an incidence of 0.2–10% in primary surgery [1,2] and up to 28% in revision cases [3,4]. It is still the first reason for early THA revision and the second reason for revision at any time [5,6,7,8]. Particularly, dislocation is the most frequent complication when THA is performed to treat displaced fractures of the femoral neck [9]. Dislocation increases mortality in elderly patients undergoing THA, with a mortality rate of 65% compared to 10% without this complication [10]. THA instability has a multifactorial etiology, and its treatment might be challenging. Patient-related risk factors include old age, female gender, previous hip surgery, neurological diseases (e.g., dementia and Alzheimer’s), neuro-muscular diseases (e.g., Parkinson’s, stroke sequelae and myopathies), spino-pelvic abnormalities (e.g., previous spinal disorders and surgery), cognitive impairment during hospitalization and the pathology for which surgery is indicated, namely hip fractures [11,12].

Different design solutions have been proposed and adopted to decrease the risk of instability: liner augmentation wedges, angle-bore acetabular components, jumbo heads and constrained liners have been used with limited success.

Nowadays, there is increasing interest in dual mobility cups (DMCs) as an effective system to prevent dislocations in THA. DMCs are characterized by a mobile polyethylene liner, providing a double articular interface between a small head and the liner, and between the liner and a metallic acetabular cup, ensuring greater range of motion and decreasing the risk of dislocation. The first model of DMC was developed in France by Bousquet in 1974. DMCs have been used in Europe for more than 40 years, while the FDA approved their use in the USA only in 2009.

The aim of this study is to compare the short-term clinical and radiological outcomes between standard and DMC total hip replacements in two groups of patients treated for femoral neck fractures (FNF). The hypothesis is that DMC implants have the same outcomes but a lower dislocation rate in comparison to standard implants.

## 2. Materials and Methods

This retrospective, single-center study was performed on a series of 56 patients treated with THA for FNF in a three-year period (January 2017–December 2019). Half of the patients underwent THA with an uncemented standard unipolar cup (TMT^®^, Zimmer Biomet, Warsaw, IN, USA) with a 10° polyethylene liner augmentation wedge (SC group), while an uncemented DMC (Dualis^®^, Bioimpianti, Peschiera Borromeo, Italy) was used in the remaining 28 patients (DMC group). A cemented femoral stem was used in all patients (Versys Heritage^®^, Zimmer Biomet for SC group; Korus^®^, Bioimpianti for DMC group). In the SC group, a 32 mm metallic femoral head was used, while DMCs were coupled with a 28 mm ceramic head.

Preoperative planning was performed on digital radiograms of the contralateral hip with the use of OrthoView software for Carestream PACS (Carestream Health, Rochester, NY, USA).

All the procedures were performed through a postero-lateral approach, with reattachment of the short external rotators. A double administration of vancomycin was used for antibiotic prophylaxis: 1 g preoperatively and 0.5 g 12 h after surgery.

In-hospital rehabilitation was started the first day after surgery and patients were mobilized out of bed on day 2 for gait re-education with a walking frame.

After discharge from the hospital, patients were evaluated clinically and radiographically at 1, 3, 6 and 12 months after surgery. At the last follow up, the WOMAC osteoarthritis index [13] was recorded.

Acetabular osteointegration was analyzed on X-rays using the Moore criteria [14]: the presence of the three most sensitive signs (absence of radiolucent lines, presence of supero-lateral buttress and presence of medial stress-shielding) defined the cup as “osteointegrated”.

Statistical analysis was performed with STATA 13 software (StataCorp LLC, College Station, TX, USA). The scores were compared with the use of a paired Student’s *t*-test for parametric data and with the use of Wilcoxon–Mann–Whitney test for non-parametric data. The significant cut-off for the *p*-value was set to 0.05. We compared the two groups for demographic data, comorbidities, length of stay from surgery to discharge, dislocation and infection rate, osteointegration, heterotopic ossification and WOMAC score. We also investigated correlations between WOMAC and each variable in both groups and in the total population of the study.

## 3. Results

At an average follow up of 23 months (range, 12 to 40), 48 patients were evaluated with the WOMAC score. Eight patients (four in each group) died for reasons not related to surgery before the last follow up: no postoperative complications were recorded among these patients, who were eventually excluded for further analyses. Thus, 24 patients for each group were included in the study and fully assessed.

Demographic and relevant clinical data of the patients are reported in Table 1. The two groups of patients were comparable for age and gender. The analysis of comorbidities revealed a statistically significant prevalence of neurologic comorbidities and psychiatric disorder in DMC group.

The mean length of hospital stay was 8.14 days (SD ± 3.18) for the DMC group and 7.75 (SD ± 3.19) for the SC group; the difference was not statistically significant (*p*-value = 0.64).

**Figure 1:**
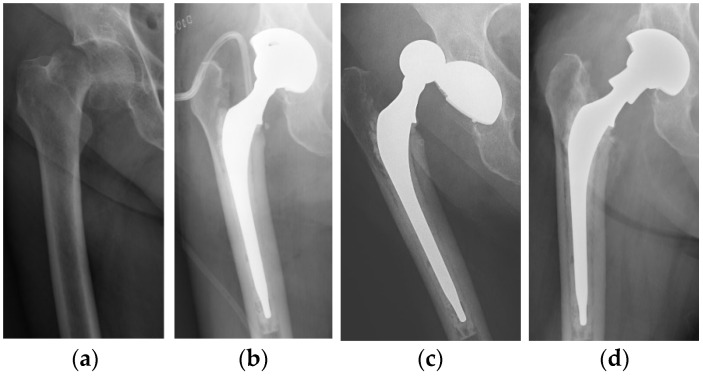
(**a**) Displaced intracapsular fracture (Garden-type IV) of the right femoral neck in a 79-year-old woman. (**b**) Postoperative radiograph after surgical treatment with a conventional THA. (**c**) Prosthesis dislocation 3 weeks after surgery. (**d**) Radiograph after implantation of a neck adaptor to increase length and lateral offset.

No dislocation occurred in the DMC group, while two dislocations were observed in the SC group. Both dislocations occurred during the first month after surgery: one in a male patient following an accidental fall and one in a female patient without any traumatic event. The first patient was treated with closed reduction and THA dislocation never recurred. The female patient suffered of postoperative moderate-grade delirium with poor cooperation in the rehabilitation program; dislocation occurred twice in the rehabilitation institute. In consideration of her mental state and poor compliance, she underwent a revision procedure: the femoral stem and acetabular cup were retained, the liner and the head were substituted and a neck adapter (Bioball^®^, Merete GmbH, Berlin, Germany) was implanted to increase length and lateral offset in order to enhance implant stability (Figure 1). Dislocation did not recur and at follow up the patient reported complete recovery of autonomy and preoperative activities.

No other complications, such as infections or mechanical failures, were observed in this series of patients.

At follow up, the mean WOMAC score for all patients was 6.26 (range, 0–46). The score in the DMC group was better (lower) than in the SC group: 4.94 (SD ± 9.12) vs. 7.58 (SD ± 12.5). However, the difference was not statistically significant (*p*-value = 0.41).

**Figure 2:**
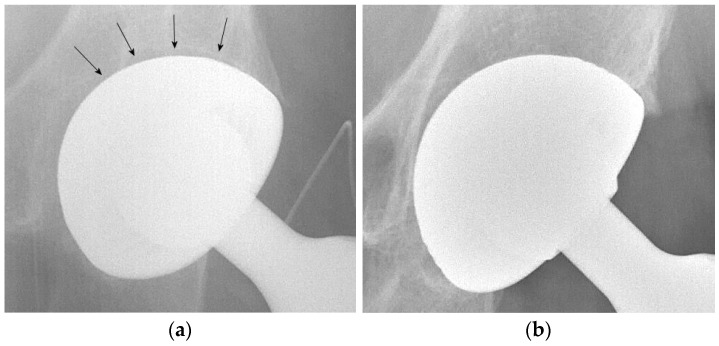
(**a**) Postoperative radiograph of a DMC showing (arrows) a radiolucent line at the bone-implant interface. (**b**) Radiographic evidence of implant osteointegration one year after surgery.

Radiographic signs of acetabular osteointegration at 1 year (Figure 2) were found in 42 patients (87.5%): 20 in the DMC group (83.3%) and 22 in the SC group (91.6%), with a non-significant difference (*p*-value = 0.98). No cases of implant mobilization were detected on X-rays. Heterotopic ossifications had a similar incidence in the two groups: four in the DMC group and three in the SC group (*p*-value = 0.99).

Correlations between the WOMAC score and clinical-radiographic variables in the total population are shown in Table 2.

## 4. Discussion

The dual mobility concept was developed by Bousquet 50 years ago to decrease the risk of THA dislocation. In standard THA, it was demonstrated that head sizes larger than 36 mm increase the head/neck ratio and the “jumping” distance. Consequently, impingement between neck and the liner rim is reduced and hip stability is increased [15]. The presence of two distinct articulations in the DMC combines Charnley’s principle of low friction arthroplasty with the McKee–Farrar concept of larger femoral heads to enhance stability: primary movement occurs at the inner bearing, while the outer bearing only moves at the extreme ranges of movement [16]. Owing to these features, the use of DMCs for the treatment of FNF has increased exponentially in the last decade [17].

Several trials comparing THA and hemiarthroplasty (HA) for the treatment of FNF showed better functional outcomes and lower re-operation rates for THA, but also a higher incidence of dislocations [18,19]. In literature, good results with DMC implants were reported in either FNF [20,21,22] or other conditions at increased risk for dislocation [10]. However, there are few retrospective and some register studies comparing DMC THA and standard THA in FNF [17,23,24,25], despite it being known that THA for fractures has a higher risk of dislocation than THA for osteoarthritis [26,27,28]. This risk could be related to different predisposing conditions observed in FNF patients, such as a good preoperative hip ROM, the prevalence of recurrent falls in older patients or the frequent postoperative delirium that compromises adherence to rehabilitation programs [29,30]. This increased risk is further confirmed by some systematic reviews: the authors of [31] reported that DMC THA is associated with a significantly lower dislocation rate compared with conventional THA (OR 0.26; 95% CI, 0.08 to 0.79); this was also confirmed by Romagnoli M. et al. [32], who showed a slight significant risk ratio of 0.16 (95% CI, 0.09, 0.28; I^2^ = 0%, *p*-value < 0.00001), with a statistically significant difference between the two groups.

The one-year mortality rate after FNF reported in the literature ranges between 14% and 36% [33,34,35]. This incidence is mainly influenced by patient-related factors, which include age, systemic comorbidities, short-term complications and mental and motivational states [36]. However, the quality of health care and the promptness of rehabilitation are also critical factors to improve the prognosis of these patients. The mortality rate in this series of patients (mean age 77.7 years) was 14.3% at one year, in accordance with the data reported by Tarasevicius et al. in similar studies [23,24].

We reported a dislocation rate of 8.3% (2/24) in the SC group, while no dislocation was observed in the DMC group. The difference did not reach a statistical significance, probably because the sample size was too small. However, it is worth to underline that patients affected by neuromuscular disease, such as Parkinson’s disease and hemiparesis following a stroke, were statistically predominant in the DMC group. This observation supports the use of DMCs in conditions with a higher risk of THA dislocation.

Tarasevicius et al. compared the results of 56 standard cups and 42 DMCs in a series of 98 patients treated with THA for FNF [23]. All the procedures were performed through a postero-lateral approach without any soft tissue repair. They reported a dislocation rate of 14.2% for the standard cups, whereas no dislocations nor intraprosthetic dislocations were reported in the DMC group; the difference was statistically significant.

Adam et al., in a multicentric study of 214 FNF patients treated with DMC THA, reported a dislocation rate of 1.4% (3 cases). All dislocations were posterior and occurred at the large articulation between the polyethylene liner and the metallic shell. They were observed within 3 months from surgery and were uneventfully treated with close reduction under general anesthesia. Poor anteversion of the cup at X-ray was noted in all these patients, who were operated on through a posterior approach [20].

Recent register studies referred for DMC THA a lower risk of revision for dislocation when compared to conventional THA [17,25].

Johansson et al. reported a dislocation rate of 22% in conventional THA performed for FNF, driving the attention to the poor adherence of these patients to postoperative prescriptions [37]. In the present series, one dislocation (standard cup) occurred in an 81-year-old woman, who lived alone and experienced postoperative delirium. The risk should be carefully assessed when choosing the type of hip arthroplasty for FNF: when THA is selected, a DMC implant seems to be more reliable in preventing dislocation for older and non-compliant (e.g., cognitively impaired) patients.

For the treatment of FNF, some authors proposed to implant the DMC through the Hueter’s anterior approach [38,39]. This surgical technique offers an additional protection against early THA dislocations, as observed with bipolar hemiarthroplasty [40]. However, there are some drawbacks for this approach when performed in older osteoporotic patients, including a higher risk of intraoperative fractures and a more troublesome cementing technique.

We did not observe any case of intraprosthetic dislocation (IPD) between the retentive polyethylene liner and the prosthetic head, but a longer follow up is needed to exclude the occurrence of this complication. IPD is a long-term complication of earlier dual mobility implants, mainly related to implant wear at the retentive rim of the polyethylene liner. Early sporadic IPD was also reported for newer implants, but they occurred after revision procedures for THA dislocation [41,42] or were consequent to assembly errors, namely poor impaction of the polyethylene inserts over the prosthetic head [21]. Tabori-Jensen et al., in a consecutive series of 966 patients treated with DMC for FNF, referred a dislocation rate of 4.7%. They reported eight IPD: six occurred during an attempt of closed reduction and two were related to a fall [43].

There are no conclusive data supporting the hypothesis that DMC are associated with a higher risk of infection. It has been hypothesized that additional manipulative maneuvers for impacting the head in the DMC liner might represent a potential source for infection [44]. A cohort study, based on the Nordic Arthroplasty Register Association (NARA) database, showed a higher risk of revision for infection for DMCs in primary THA for osteoarthritis [45]. However, the authors highlighted that this finding could be related to a selection bias, since DMCs were implanted in patients with greater frailty and therefore with a baseline increased risk of infection [45]. Match cohort study and meta-analysis of comparative study confuted this finding, reporting higher infection rates with the standard cup than with the DMC in revision procedures [46,47]. Jobory et al., in another register study from NARA database, matched 4520 hip fractures treated with DMCs to 4520 hip fractures treated with conventional THA [25]. They concluded that DMC-treated patients had a lower risk of revision in general including and no difference regarding revision for infection. We did not detect any prosthetic infection in our small series of patients, with two comparable groups for diabetes and obesity.

According to the WOMAC scores recorded (mean 6.26), the clinical outcome of the patients included in this study was good, with a non-significant superiority in favor of DMC patients. Better scores in patients with neurologic comorbidities and psychiatric disorders, which were predominant in the DMC group, seem to validate the use of DMCs in these conditions.

In a retrospective study, Fahad et al. compared DMC THA with bipolar HA for the treatment of displaced FNF in a series of 99 patients (77 BHA, 22 DMC) with a mean age of 70 years. At an average follow up of 20 months, better hip functional outcomes were observed in the DMC group (mean Harris hip score of 76.8 vs. 68.8), while no significant difference was noted in terms of postoperative surgical complications and one-year mortality rate [48]. In a study comparing DMCs and conventional THA for FNF, Tarasevicious et al. found no difference between the two groups of patients at any time of follow up, considering any subscale of HOOS score, mobility, use of walking aids and EQ-5D [48]. Adam et al., at the 9-month follow up of their multicentric study, observed that 70% of DMC-treated patients for FNF had returned home with no increase in dependency, 50% did not need any walking assistance at home and 31% were independent from walking aids [20].

Radiographic examination did not reveal any complication at 1-year follow up, with a rate of 87.5% (42/48) osteointegrated cups in the total population. The SC group showed a higher number of osteointergrated cups than DMC (22 vs. 20), but this difference was not significant. It must be noted that the DMCs used in our series did not allow for the insertion of screws to increase primary stability of the acetabular cup. This is a drawback in patients with compromised bone quality and might require cement fixation. In a recent study, Sunilkumar et al. highlighted the risk of improper cup fixation and periprosthetic acetabular fractures with the use of DMCs for FNF in elderly patients [49]. The lack of screws for fixation and the inability to visualize the acetabular floor during impaction were considered disadvantages of this implant, particularly in presence of osteoporotic bones.

## 5. Conclusions

In the past decades, concerns have been raised for the long-term survival of first-generation DMCs, since the additional bearing surface could accelerate polyethylene wear and increase the risk of aseptic loosening [50]. However, recent register studies comparing DMC and conventional THA have ruled out these concerns, reporting no differences in revision rates for loosening with newer implants [17,25].

Despite that our study was limited by a small sample size, according to our experience and to the most recent literature on the topic, we conclude that DMC THA presents short-term outcomes comparable to conventional THA. The use of DMCs for the treatment of displaced FNF in older patients is a reasonable choice, since it allows for a decrease in the risk of postoperative dislocations and improves the prognosis of these frail and often non-cooperative subjects.

## Figures and Tables

**Table 1:** Demographic and relevant clinical data of the study population.

	DMC Group *n* = 24	SC Group *n* = 24	*p*-Value
Age	77.03	78.35	0.41
Gender (M:F)	7:21	7:21	1.00
Neurological comorbidities ^(1)^	12	4	0.03 *
Diabetes	0	3	0.23
Obesity	5	2	0.41
Rheumatological diseases	2	2	0.99
Psychiatric disorders ^(2)^	6	0	0.02 *

^(1)^ Parkinson’s disease *n* = 3/1; Hemiplegia n = 1/0; Hemiparesis *n* =8/3. ^(2)^ Anxiety-depressive disorder *n* = 6/0. (*) statistically significant *p*-values.

**Table 2:** Correlations between the WOMAC score and clinical-radiographic variables at follow up.

	Yes/No	Mean WOMAC	*p*-Value
Neurological comorbidities	y 14	1.48	0.04 *
	n 34	8.23	
Diabetes	y 3	17.0	0.04 *
	n 45	5.55	
Obesity	y 7	1.56	0.23
	n 41	7.06	
Rheumatological diseases	y 4	1.25	0.32
	n 44	6.72	
Psychiatric disorders	y 6	0.00	0.02 *
	n 42	7.16	
Dislocation	y 2	6.47	0.80
	n 46	1.5	
Osteointegration	y 37	4.96	0.16
	n 7	15.5	
Heterotopic ossifications	y 7	6.70	0.22
	n 41	6.19	

WOMAC scores resulted to be significantly better in the presence of neurological comorbidities, in the absence of diabetes and in the case of psychiatric disorders. No statistical correlation could be found inside each group and in the comparison between the two groups because the sample size was too small. Better WOMAC scores, but without statistical significance, were found in the case of osteointegrated cups (*p*-value = 0.16). (*) statistically significant *p*-values.

## Data Availability

The data presented in this study are available on request from the corresponding author.
